# Long-Term Results of Medial Fixed-Bearing Unicompartmental Knee Arthroplasty with Miller-Galante Prosthesis: A Minimum 10-Year Follow-Up Study

**DOI:** 10.3390/medicina62040663

**Published:** 2026-03-31

**Authors:** Sumin Lim, Tae Hun Kim, Do Young Park, Hyun Il Choi, Jun Young Chung

**Affiliations:** Department of Orthopedic Surgery, School of Medicine, Ajou University, 164 Worldcup-ro, Yongtong-gu, Suwon 16499, Republic of Korea; khoo1003@gmail.com (S.L.); realthkim@hanmail.net (T.H.K.); doytheboy@hanmail.net (D.Y.P.); medijc77@gmail.com (H.I.C.)

**Keywords:** unicompartmental knee arthroplasty, survival, knee, osteoarthritis

## Abstract

*Background and Objectives*: Medial unicompartmental knee arthroplasty (UKA) has emerged as an effective surgical option for isolated medial compartment osteoarthritis (OA), offering advantages in bone preservation, knee kinematics, and postoperative recovery compared with total knee arthroplasty (TKA). Although numerous studies have evaluated the mid- to long-term outcomes of UKA, reports focusing on cohorts with follow-up periods exceeding 10 years remain relatively limited. The purpose of this study was to analyze the long-term clinical and radiological results of medial fixed-bearing UKA using the Miller-Galante prosthesis. *Methods*: Sixty-eight patients who underwent UKA at a single institution with at least 10 years of follow-up were retrospectively reviewed. Clinical outcomes were assessed using the Western Ontario and McMaster Universities Osteoarthritis Index (WOMAC) score and knee range of motion (ROM). Radiological parameters including the hip-knee-ankle axis angle (HKA) and osteoarthritis (OA) grade using the Kellgren-Lawrence (K-L) grading system were evaluated. Implant survivorship was evaluated using Kaplan–Meier survival analysis. *Results*: A total of 68 patients were included with a mean age of 56.8 ± 7.5 years at surgery and a mean follow-up of 170.9 ± 37.3 months. Significant improvement in the WOMAC score was observed from 48.9 ± 17.2 preoperatively to 23.8 ± 27.7 at final follow-up (*p* = 0.002). The cumulative survival rates were 97.1% at 10 years and 84.8% at 15 years with conversion to total knee arthroplasty as the endpoint. Significant improvement in the HKA was observed from 172.5° ± 4.4° to 174.3° ± 4.8° postoperatively (*p* = 0.002), though residual varus alignment persisted. Progressive OA was observed in the lateral tibiofemoral and patellofemoral compartments (both *p* < 0.001) but showed no correlation with the WOMAC score. The failure group showed trends toward higher body mass index (BMI) and smaller preoperative HKA angle compared to the non-failure group. *Conclusions*: The long-term outcomes of medial fixed-bearing UKA using the Miller–Galante prosthesis were generally favorable, with significant functional improvement and acceptable implant survivorship. Although overall varus alignment was corrected, some residual varus deformity remained, and OA progression was observed in the lateral tibiofemoral and patellofemoral compartments over time. However, given the retrospective design and limited sample size, these findings should be interpreted with caution.

## 1. Introduction

Osteoarthritis (OA) of the knee is one of the most prevalent musculoskeletal conditions worldwide, with isolated medial compartment involvement representing the most common pattern of tibiofemoral OA. Medial unicompartmental knee arthroplasty (UKA) is a well-established treatment option for isolated medial compartment OA and has demonstrated favorable clinical outcomes and implant survivorship [1,2]. Compared with total knee arthroplasty (TKA), UKA offers several advantages, including preservation of bone stock, more physiological knee kinematics, faster postoperative recovery, reduced intraoperative blood loss, and a lower incidence of perioperative complications [3,4]. However, despite these benefits, previous studies have reported that the long-term survivorship of UKA remains slightly inferior to that of TKA [5,6].

Patient selection has been recognized as a critical determinant of UKA outcomes. The traditional indications for UKA, as described by Kozinn and Scott, included isolated single-compartment OA, age older than 60 years, weight less than 82 kg, low activity demands, flexion greater than 90°, flexion contracture less than 5°, and angular deformity less than 15° [7]. These strict criteria were intended to minimize the risk of progression in the non-resurfaced compartments and implant failure. In recent years, however, these criteria have been progressively extended, and excellent outcomes have been reported in younger patients, those with higher body mass index (BMI), and even in the presence of anterior cruciate ligament deficiency when combined with ligament reconstruction [8]. Among the two primary UKA designs, mobile-bearing and fixed-bearing, each has distinct biomechanical characteristics. Mobile-bearing designs allow rotational movement of the polyethylene insert, theoretically reducing contact stress and wear, but bearing dislocation can occur as a potential complication. Fixed-bearing designs, by contrast, offer greater constraint and simplified implant mechanics, eliminating the risk of bearing dislocation. Several studies have demonstrated that fixed-bearing prostheses show comparable or even superior survivorship and long-term stability compared with mobile-bearing designs [9,10,11,12].

Although numerous studies have evaluated the mid- to long-term outcomes of UKA, reports focusing on cohorts with follow-up periods exceeding 10 years remain relatively limited. Therefore, the purpose of this study was to evaluate the long-term clinical and radiographic outcomes of medial fixed-bearing UKA using the Miller-Galante prosthesis performed at a single institution with a minimum follow-up of 10 years. Additionally, factors associated with implant failure and revision were analyzed. It was hypothesized that medial fixed-bearing UKA using the Miller-Galante prosthesis would demonstrate satisfactory long-term clinical and radiological outcomes, with acceptable implant survivorship over a minimum 10-year follow-up period.

## 2. Materials and Methods

### 2.1. Study Design and Ethical Approval

This retrospective cohort study received approval from the Institutional Review Board of our hospital (AJOUIRB-DB-2026-056). Given the retrospective design, the requirement for informed consent was waived.

### 2.2. Patient Selection

Between March 2002 and December 2012, a total of 206 primary medial UKAs were performed at our institution, of which 160 used the Miller-Galante fixed-bearing prosthesis (Zimmer, Warsaw, IN, USA) and were considered for inclusion. Inclusion criteria were: (1) primary medial compartment knee OA, (2) minimum 10-year follow-up, and (3) adherence to established surgical indications including coronal deformity less than 15°, flexion contracture less than 15°, knee flexion greater than 90°, intact anterior and posterior cruciate ligaments, competent collateral ligaments, and absence of inflammatory arthritis. No restrictions were applied regarding age or BMI. Exclusion criteria comprised: inflammatory arthropathy (including rheumatoid arthritis, crystal arthropathy such as gout and calcium pyrophosphate deposition disease, and other rheumatic disorders); post-traumatic OA; ligamentous insufficiency or lateral meniscal pathology; lateral tibiofemoral OA (Kellgren-Lawrence (K-L) grade 3 or higher); patellofemoral OA (K-L grade 3 or higher).

### 2.3. Surgical Technique and Rehabilitation Protocol

All operations were performed by a single senior surgeon with 15 years of experience in knee arthroplasty, with UKA comprising more than 20% of the surgeon’s annual arthroplasty volume. The surgical procedure followed standardized protocols [13]. Through a medial parapatellar approach, intraoperative assessment of articular cartilage confirmed isolated medial compartment OA, consistent with the preoperative radiological findings. The Zimmer Miller-Galante fixed-bearing UKA system was used in all cases. The surgical technique adhered to the manufacturer’s instrumentation protocol using conventional instruments. The tibial resection was performed first, perpendicular to the mechanical axis using an extramedullary alignment guide. Femoral preparation was performed using dedicated cutting guides. After balancing flexion and extension gaps, the appropriate polyethylene insert thickness was selected. All components were fixed with bone cement. Postoperative rehabilitation was initiated on day 1 with continuous passive motion from 0° to 60°, progressing as tolerated; full weight-bearing ambulation with assistive devices was permitted from the first postoperative day.

### 2.4. Outcome Assessment

Patients underwent clinical evaluation and radiographic examination, including standing anteroposterior, lateral, and Merchant views of the knee, along with full-leg standing radiographs. Assessments were conducted preoperatively, at 1, 3, and 6 months postoperatively, and annually thereafter. For patients who did not attend scheduled clinic visits, telephone surveys were conducted to assess clinical outcomes and determine whether revision or conversion to TKA had occurred.

Primary outcomes were the long-term clinical and radiological outcomes after medial UKA. Clinical outcomes included the Western Ontario and McMaster Universities Osteoarthritis Index (WOMAC), knee range of motion (ROM), and implant survivorship. Failure was defined as conversion to total knee arthroplasty. Implant survivorship was calculated using Kaplan–Meier survival analysis. Radiological outcomes included the hip-knee-ankle angle (HKA) and OA progression in the lateral and patellofemoral compartments using the K-L classification system: grade 1 (doubtful joint space narrowing with possible osteophytic lipping), grade 2 (definite osteophytes with possible joint space narrowing), grade 3 (moderate osteophytes, definite joint space narrowing, sclerosis, and possible bone deformity), and grade 4 (large osteophytes, marked joint space narrowing, severe sclerosis, and definite bone deformity) [14]. All radiographic measurements were performed using the Picture Archiving and Communication System (PACS; Carestream Health, Rochester, NY, USA). Knee ROM was assessed by measuring passive full extension and full flexion angles using a goniometer, with arc of motion calculated as the difference between the extension and flexion angle.

Secondary outcomes included the correlation between clinical and radiological outcomes and identification of risk factors by comparing patient and surgical characteristics between the failure and non-failure groups.

### 2.5. Statistical Analysis

Continuous variables were compared between groups using independent samples *t*-tests after confirming normality with the Shapiro–Wilk test. Categorical variables were analyzed using chi-square or Fisher’s exact test as appropriate. Correlations were assessed using Pearson’s correlation coefficient (r). All statistical analyses were performed using R software (version 3.6.3; R Foundation for Statistical Computing, Vienna, Austria), with statistical significance set at *p* < 0.05.

## 3. Results

Of the 160 patients, 6 were excluded due to periprosthetic fracture (n = 2) or concomitant ACL reconstruction (n = 4), and 86 were lost to follow-up or died prior to the 10-year minimum follow-up period; the remaining 68 patients were included in the final analysis. The mean age at the time of surgery was 56.8 ± 7.5 years, and the mean follow-up duration was 170.9 ± 37.3 months (Table 1).

Significant improvement in the WOMAC score was observed from baseline (48.9 ± 17.2) to final follow-up (23.8 ± 27.7, *p* = 0.002). Knee extension improved significantly from 1.7° ± 3.8° preoperatively to 0.3° ± 1.5° at final follow-up (*p* = 0.014), whereas knee flexion (151.0° ± 20.4° vs. 154.0° ± 11.6°, *p* = 0.358) and arc of motion (149.4° ± 21.1° vs. 153.7° ± 12.3°, *p* = 0.203) showed no significant change.

**Figure 1:**
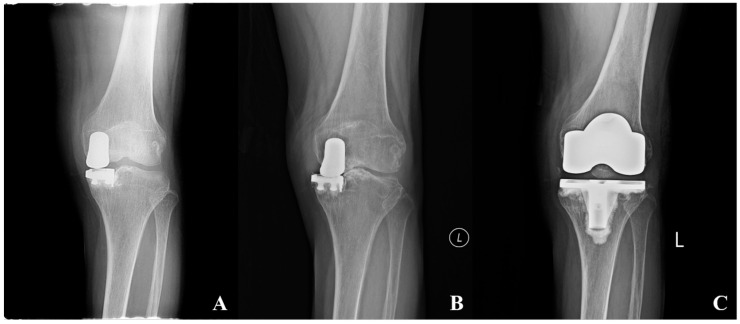
Revision to total knee arthroplasty for polyethylene wear and osteoarthritis progression after medial fixed-bearing unicompartmental knee arthroplasty (UKA). A 64-year-old female patient who underwent revision total knee arthroplasty (TKA) following medial fixed-bearing UKA in the left knee. (**A**) Postoperative anteroposterior radiograph after medial fixed-bearing UKA. (**B**) Knee anteroposterior radiograph at 13.3 years postoperatively demonstrating polyethylene wear and osteoarthritis progression. (**C**) Postoperative anteroposterior radiograph after revision to TKA. The revision was performed as a primary TKA without the use of stems or metal blocks.

**Figure 2:**
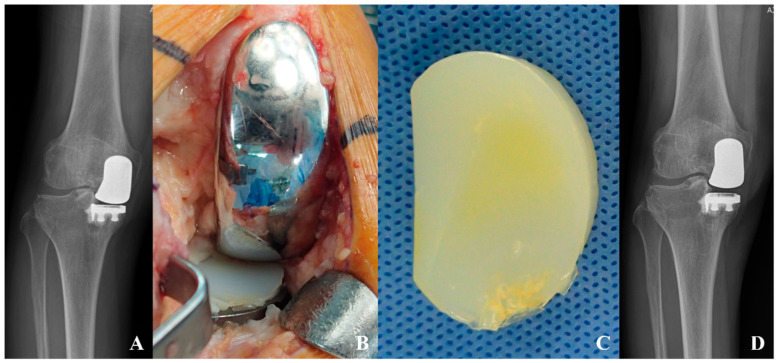
Isolated polyethylene insert exchange for polyethylene wear after medial fixed-bearing unicompartmental knee arthroplasty (UKA). A 57-year-old female patient who underwent medial fixed-bearing UKA in the right knee. The patient remained asymptomatic for 8 years postoperatively but developed vague pain on knee bending that persisted for 2 years. (**A**) Plain radiographs showing slight varus alignment with asymmetric narrowing between femoral and tibial components. (**B**,**C**) Intraoperative findings demonstrating polyethylene wear on the anteromedial aspect. Both femoral and tibial components remained stable and well-fixed. (**D**) Postoperative radiographs after isolated polyethylene insert exchange performed at 9.8 years after the initial surgery.

**Figure 3:**
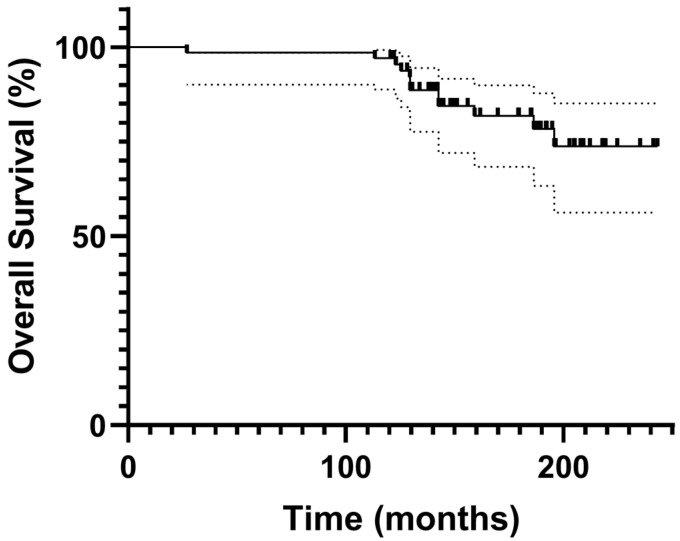
Kaplan–Meier survival curve for medial unicompartmental knee arthroplasty using the Miller-Galante fixed-bearing design. The cumulative survivorship at 10 and 15 years was 97.1% and 84.8%, respectively. Gray dots represent 95% confidence intervals.

Twelve patients (17.6%) underwent reoperation during the follow-up period. Ten cases (14.7%) were classified as failures requiring conversion to total knee arthroplasty: two (2.9%) due to progressive OA in untreated compartments, one (1.5%) due to aseptic loosening, and seven (10.3%) who underwent conversion at outside institutions with unspecified indications (Figure 1). Two additional patients (2.9%) underwent isolated polyethylene insert exchange due to polyethylene insert wear in the setting of well-fixed implant components and therefore were not classified as failures (Figure 2). Kaplan–Meier survival analysis demonstrated a 10-year survivorship of 97.1% and 15-year survivorship of 84.8%, with conversion to total knee arthroplasty as the endpoint (Figure 3).

**Figure 4:**
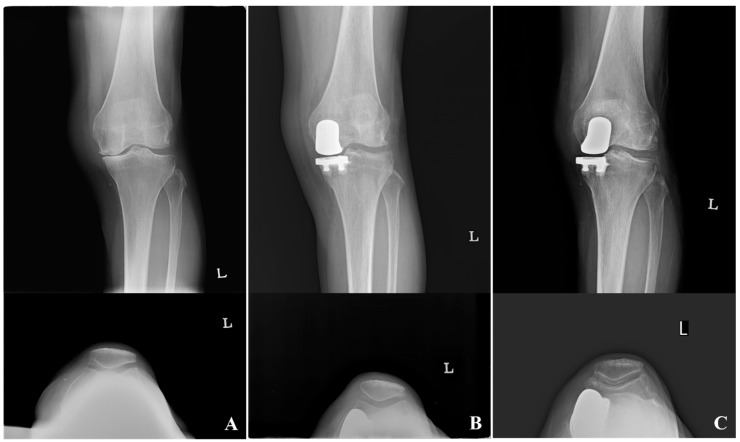
Excellent clinical outcome despite radiographic osteoarthritis progression at a 17-year follow-up after medial fixed-bearing unicompartmental knee arthroplasty (UKA). A 54-year-old female patient who underwent medial fixed-bearing UKA in the left knee. (**A**) Preoperative knee anteroposterior and merchant radiographs showing medial osteoarthritis. (**B**) Postoperative knee anteroposterior and merchant radiographs after medial fixed-bearing UKA. (**C**) Knee anteroposterior and merchant radiographs at 17 years postoperatively demonstrating osteoarthritis progression in the lateral tibiofemoral and patellofemoral compartments. Despite radiographic deterioration, the patient showed excellent clinical outcomes with a WOMAC score of 7.

The HKA improved significantly from preoperative values (172.5° ± 4.4°) to postoperative alignment (174.3° ± 4.8°, *p* = 0.002), although a mean residual varus of approximately 5.7° persisted. Progressive OA in the lateral (*p* < 0.001) and patellofemoral compartments (*p* < 0.001) were observed during the minimum 10-year follow-up period (Table 2, Figure 4). However, no significant correlation was found between postoperative OA grade or progression and WOMAC scores (Table 3).

Comparison between the failure group (n = 10) and non-failure group (n = 58) revealed trends toward higher BMI (28.3 ± 4.9 vs. 25.4 ± 2.9 kg/m^2^, *p* = 0.09) and smaller preoperative HKA (170.0° ± 4.0° vs. 172.9° ± 4.4°, *p* = 0.065) in the failure group, though neither reached statistical significance. Age was similar between the two groups (54.3 ± 6.9 vs. 57.2 ± 7.6 years, *p* = 0.253).

## 4. Discussion

The long-term clinical, radiological, and survivorship outcomes of medial fixed-bearing UKA using the Miller-Galante prosthesis were evaluated in this study. The 10- and 15-year survivorship rates were 97.1% and 84.8%, respectively, with generally favorable clinical outcomes observed despite residual mild varus and radiographic OA progression. These findings are consistent with current evidence suggesting that slight undercorrection in medial UKA is acceptable and may still yield favorable functional results [15,16]. Moreover, several long-term series have reported that radiographic progression in the non-operated compartments does not necessarily correlate with deterioration in patient-reported outcomes [17,18]. In this regard, the present study adds to the growing body of evidence that long-term implant durability should be interpreted together with patient-reported function rather than by survivorship alone.

Several studies of fixed-bearing medial UKA have reported 10-year survivorship ranging roughly from 89% to 98% and a gradual decline thereafter with longer follow-up [19,20,21,22]. For example, Calkins et al. observed a survivorship of 90.4% at 10 years and 75.1% at 19 years in patients younger than 55 years undergoing fixed-bearing medial UKA [22]. Hung et al. reported survivorship rates of 91.6% at 10 years and 90.0% at 15 years for a fixed-bearing medial design [21]. Mannan et al. similarly reported 10- and 15-year survivorship rates of 92.9% and 87.8%, respectively, in a cohort treated with a fixed-bearing medial design [19]. John et al. demonstrated survivorship rates of 94% at 10 years and 87% at 15 years using the Miller-Galante prosthesis [20]. In the present study, medial fixed-bearing UKA using the Miller-Galante prosthesis showed 10- and 15-year survivorship of 97.1% and 84.8%, respectively, which falls within this reported range for fixed-bearing designs. By contrast, registry-based studies of TKA have demonstrated 15- and 20-year survivorship commonly exceeding 93% and 90%, respectively, indicating that TKA still achieves superior long-term implant survival compared with UKA [23]. Recent meta-analyses have consistently demonstrated that TKA exhibits lower revision rates and superior long-term survivorship compared to UKA [5,24]. With respect to patient-reported outcomes, the WOMAC scores observed in the present study are comparable to those reported in previous long-term series of medial UKA. In the present study, the mean WOMAC score improved significantly from 48.9 ± 17.2 preoperatively to 23.8 ± 27.7 at a mean follow-up of 170.9 months, representing a clinically meaningful improvement in functional outcomes. David et al. reported mean WOMAC scores of approximately 25.1 at 10 years and 19.5 at 12 years following medial UKA [25]. Similarly, Forster et al. found a mean total WOMAC score of 23.1 at 8 years, which aligns closely with our findings [26]. Meta-analyses directly comparing UKA and TKA have demonstrated that UKA tends to yield superior functional outcomes and activity-related patient-reported scores, despite similar pain relief, supporting the clinical advantages of UKA in preserving knee function [5]. These results indicate that the functional benefit of UKA may remain meaningful even when long-term survivorship is lower than that of TKA.

Significant improvement in the HKA was observed after surgery (172.5° ± 4.4° to 174.3° ± 4.8°, *p* = 0.002), with a mean residual varus of approximately 5.7° remaining at final follow-up without compromising clinical results. A mild residual varus (approximately 2–7°) after medial UKA is not detrimental and may even be associated with superior functional outcomes compared with neutral or near-neutral alignment [15,16]. Restoring a slight varus closer to the patient’s pre-arthritic alignment may optimize load distribution and kinematics in medial UKA, and the favorable WOMAC scores observed in the present study despite undercorrection support this concept [27]. Residual varus after UKA therefore does not necessarily indicate inadequate correction but may reflect a balance between mechanical alignment and preservation of native knee kinematics. In the present study, the failure group exhibited a trend toward greater preoperative varus deformity, although this did not reach statistical significance. Similar trends have been reported in other studies, where more severe preoperative varus was associated with increased risk of suboptimal alignment correction and early implant failure [28]. Significant improvement in knee extension was observed after surgery (1.7° ± 3.8° to 0.3° ± 1.5°, *p* = 0.014), whereas knee flexion and total arc of motion showed no statistically significant change. Although the degree of preoperative flexion contracture was minimal in our cohort, its significant improvement at long-term follow-up suggests that medial fixed-bearing UKA effectively resolves mild contracture without compromising overall ROM. Consistent with these findings, Wignadasan et al. reported that flexion contracture improved following UKA regardless of its preoperative severity [29]. Furthermore, Goh et al. reported that even in patients with preoperative flexion contracture greater than 15°, residual contracture and poorer ROM persisted postoperatively, but patient-reported outcome measures and implant survivorship were not significantly different from those without contracture [30].

The most common causes of revision following fixed-bearing medial UKA include aseptic loosening and OA progression in the non-resurfaced compartments [31]. However, the presence of radiographic OA progression does not necessarily indicate the need for revision, as long-term UKA series have consistently reported OA progression in the non-resurfaced compartments without a corresponding deterioration in clinical outcomes. In the present study, significant OA progression was observed in both the lateral tibiofemoral (*p* < 0.001) and patellofemoral compartments (*p* < 0.001) at a mean follow-up of 170.9 months. Similar observations have been reported in other long-term UKA studies. For example, Walker et al. noted radiographic progression in the lateral compartment in approximately 40% of patients at extended follow-up, but functional outcomes remained satisfactory [18]. Likewise, Faour Martín et al. observed degenerative changes in the lateral and patellofemoral compartments that did not correlate with symptomatic decline or necessitate revision surgery [17]. Notably, however, OA progression in either compartment did not correlate with WOMAC score deterioration in the present study, suggesting that radiographic deterioration does not necessarily translate into clinical decline. Radiographic OA progression after UKA should therefore be interpreted with caution, and surgical intervention should not be based on radiographic findings alone in the absence of corresponding clinical symptoms.

The influence of obesity and age on UKA outcomes remains controversial in the literature. Elevated BMI may increase mechanical loading on the implant, potentially accelerating polyethylene wear and contributing to early implant failure. Some reports have linked obesity to higher revision rates or early failure in UKA [32], while others found no significant adverse effect of elevated BMI on survivorship or clinical outcomes [33,34]. Regarding age, several meta-analyses and national registry data have reported higher revision rates in patients younger than 60 years [35,36]. Younger patients tend to have higher activity levels and longer life expectancy, which may increase the cumulative mechanical demand on the implant over time and contribute to higher revision rates in this population. However, other meta-analyses have concluded that UKA is a safe and reliable treatment option in patients under 60 years and should not be considered a contraindication [37,38]. In the present study, higher BMI showed a trend toward increased failure, but this association did not reach statistical significance. Age did not clearly differentiate the failure and non-failure groups. Notably, the mean age of our cohort was 56.8 years, indicating that UKA was performed in a relatively young patient population, yet acceptable survivorship and functional outcomes were achieved at long-term follow-up. Given the ongoing debate surrounding these factors, BMI and patient age should be carefully considered during preoperative patient selection and planning, and patients with elevated BMI or younger age may warrant closer postoperative surveillance to optimize long-term outcomes following medial fixed-bearing UKA.

### Study Limitations and Strengths

This study has several limitations. First, the sample size was relatively small, and a formal power analysis was not performed given the retrospective nature of the study. The limited sample size may have reduced statistical power, potentially limiting the ability to detect statistically significant associations between patient factors (BMI, age, preoperative varus) and failure. Second, causes of failure in patients who underwent revision at outside institutions could not always be clearly identified. Third, radiographic assessment was limited to HKA and compartmental OA progression; component-specific parameters such as tibial component alignment and femoral component alignment angle were not systematically evaluated, which may limit the interpretation of alignment-related outcomes. Fourth, the inclusion of only patients with a minimum 10-year follow-up may introduce selection bias, as patients with earlier failure or those lost to follow-up prior to 10 years were not captured in the final analysis. Fifth, as a single-center retrospective study, the generalizability of these findings to other institutions, implant systems, and surgical techniques may be limited. Nevertheless, the use of a single fixed-bearing design implanted by a single surgeon minimizes heterogeneity related to surgical technique and implant selection, allowing for a more homogeneous assessment of long-term outcomes.

## 5. Conclusions

The long-term outcomes of medial fixed-bearing UKA using the Miller–Galante prosthesis were generally favorable, with significant functional improvement and acceptable implant survivorship. Although overall varus alignment was corrected, some residual varus deformity remained, and OA progression was observed in the lateral tibiofemoral and patellofemoral compartments over time. However, given the retrospective design and limited sample size, these findings should be interpreted with caution.

## Figures and Tables

**Table 1:** Demographic details of the patients.

Variables	
Age (years)	56.8 ± 7.5
Sex, male/female	13/55
Height (cm)	158.2 ± 7.4
Weight (kg)	64.7 ± 10.5
BMI (kg/m^2^)	25.8 ± 3.4
Follow-up (months)	170.9 ± 37.3

BMI, body mass index.

**Table 2:** Clinical and radiological outcomes between preoperative and last follow-up.

	Preoperative	Last Follow-Up	*p*-Value
WOMAC	48.9 ± 17.2	23.8 ± 27.7	0.002
Knee extension (°)	1.7 ± 3.8	0.3 ± 1.5	0.014
Knee flexion (°)	151.0 ± 20.4	154.0 ± 11.6	0.358
Knee arc of motion (°)	149.4 ± 21.1	153.7 ± 12.3	0.203
HKA (°)	172.5 ± 4.4	174.3 ± 4.8	0.002
Lateral OA grade (0/1/2/3/4)	13/49/6/0/0	0/31/29/4/4	<0.001
Patellofemoral OA grade (0/1/2/3/4)	10/51/7/0/0	0/33/26/9/0	<0.001

WOMAC, Western Ontario and McMaster Universities Osteoarthritis Index; HKA, hip-knee-ankle angle; OA, osteoarthritis.

**Table 3:** Correlation analysis between postoperative osteoarthritis grade or progression and WOMAC scores.

Variables	Correlation Coefficient (r)	*p*-Value
Lateral OA grade vs. WOMAC score	−0.13	0.309
Lateral OA progression vs. WOMAC score	−0.10	0.452
Patellofemoral OA grade vs. WOMAC score	−0.10	0.437
Patellofemoral OA progression vs. WOMAC score	−0.08	0.544

OA, osteoarthritis; WOMAC, Western Ontario and McMaster Universities Osteoarthritis Index.

## Data Availability

Data are unavailable due to privacy or ethical restrictions.
